# Heat Transfer Enhancement of n-Type Organic Semiconductors by an Insulator Blend Approach

**DOI:** 10.1021/acsami.2c05503

**Published:** 2022-06-23

**Authors:** Zhuoqiong Zhang, Yabing Tang, Yunfan Wang, Zixin Zeng, Run Shi, Han Yan, Sai-Wing Tsang, Chun Cheng, Shu Kong So

**Affiliations:** † Department of Physics and Institute of Advanced Materials, Hong Kong Baptist University, Kowloon Tong, Hong Kong SAR 999077, P. R. China; ‡ Department of Materials Science and Engineering, Southern University of Science and Technology, Shenzhen 518055, P. R. China; § State Key Laboratory for Mechanical Behavior of Materials, Xi’an Jiaotong University, Xi’an 710049, P. R. China; ∥ Department of Materials Science and Engineering, City University of Hong Kong, Kowloon Tong, Hong Kong SAR 999077, P. R. China

**Keywords:** insulator blends, thermal stability, heat transfer, thermal transport, thermal management, organic field-effect transistor, n-type organic semiconductors

## Abstract

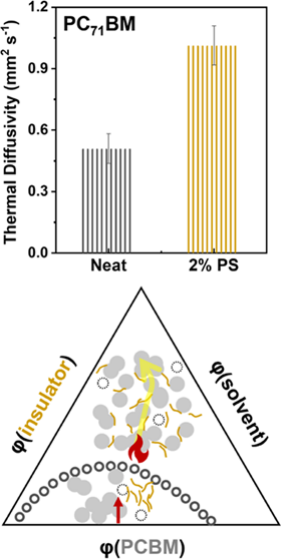

The transfer of heat energy in organic semiconductors (OSCs) plays an important role in advancing the applications of organic electronics, especially for lifetime issues. However, compared with crystalline inorganic semiconductors, the thermal transport of OSCs is less efficient and a relevant understanding is very limited. In this contribution, we show that the heat conduction of OSCs can be enhanced by blending with a “commodity” insulator (both thermal and electrical). PC_71_BM, a well-known electron transporter but poor thermal conductor, was selected as the host OSC material. The blending of a small amount of polystyrene (PS), a commonly used insulating polymer, can facilitate the heat transfer of PC_71_BM films, as substantiated by the scanning photothermal deflection technique and an infrared thermal camera. The phase thermodynamics of PC_71_BM/PS blends indicates that the efficient heat transfer preferably occurs in the OSC/insulator blends with better intimate mixing, where isolated PC_71_BM domains can be effectively bridged by PS that thread through the regions. The applicability of this approach can be observed in blends with another host material—ITIC. This work provides a facile strategy for designing thermally durable organic electronic devices.

## 1 Introduction

Organic semiconductors (OSCs) possess many attractive attributes and have therefore penetrated various electronic applications, such as organic photovoltaics (OPVs), organic field-effect transistors (OFETs), and organic light-emitting diodes (OLEDs). These applications have made steady progress. Particularly, high charge carrier mobility over 10 cm^2^ V^–1^ s^–1^ has been achieved in OFET applications.^1^ However, at a fixed device voltage, increased carrier mobility in an OFET generates a larger electrical current and Joule heating effect, leading to ineluctable thermal degradation of the device during operation. Worse, the thermal conductivity of existing OSCs, especially amorphous OSCs, is in a very low range below 1 W m^–1^ K^–1^ due to structural disorders and weak molecular interactions.^2−4^ This undesirable thermal conductivity is extremely unfavorable for heat dissipation in devices, but relevant studies to alleviate this problem are very lacking.

One way of circumventing the poor thermal conductivity in OSCs is a blending approach. In general, blending high thermal conductivity fillers, e.g., metal and ceramic particles, in OSC materials has been considered a desirable strategy.^5−7^ The fillers serve as the heat dissipation materials to release the heat accumulated during the device operation, thereby constructing thermally durable devices. However, this approach poses challenges for scaled-up applications due to the high material cost and the risks of degrading electrical and optical properties.^8,9^ Recently, Gumyusenge et al. found that blending insulators into OSCs was beneficial to the thermal stability of devices, which facilitated a high-mobility operation at up to 220 °C.^10^ This stems from the fact that the conformational change of OSCs under heating is restricted by the insulator. However, comprehensive studies on the heat transfer effect of OSC after incorporating insulators were rarely explored; the corresponding mechanism is still unclear. Hence, it is highly desirable to systematically study the thermal properties of the blended system and disclose the role of insulators in the blends for realizing efficient heat transfer.

Here, we chose [6,6]-phenyl-C71-butyric acid methyl ester (PC_71_BM) as the host OSC. PC_71_BM is known to have very poor heat conduction properties in the condensed phase due to severe vibrational scattering of electrons by its molecular tail.^11−15^ Yet, it is probably the most popular electron transporter used in organic electronics. Into PC_71_BM, we blended a small amount of one of the most easily accessible insulators, polystyrene (PS). It is found that the addition of PS can enhance the thermal stability of the as-prepared OFETs. The device reproducibility is improved while suppressing leakage current in the off-state (*I*_off_) and subthreshold swing (*SS*). The effect of insulators on heat transfer was then examined using the scanning photothermal deflection (SPD) technique and an infrared thermal imaging camera (IR camera), where the thermal diffusivity measured by SPD characterizes the rate of heat dissipation. SPD shows that the thermal diffusivity of a blended film is doubled to 1.01 mm^2^ s^–1^ when compared with a neat PC_71_BM film. Furthermore, the application of blends in electron transport layers of perovskite solar cells confirms the potential applicability of the insulator blend approach. Finally, the ternary phase diagrams comprising PS with varied molecular weights (MWs) were calculated to understand the underlying mechanism for the role of insulators in the mixtures. The better mixing of OSC and low-MW insulator allows for a more interconnected phonon transport network, thereby facilitating heat propagation. This work not only offers an understanding of heat transfer in OSC/insulator blends but also enriches the development of OSCs with promising stability for various electronic applications.

## 2 Results and Discussion

### 2.1 OFET Characteristics

#### 2.1.1 Electrical Properties

**Figure 1:**
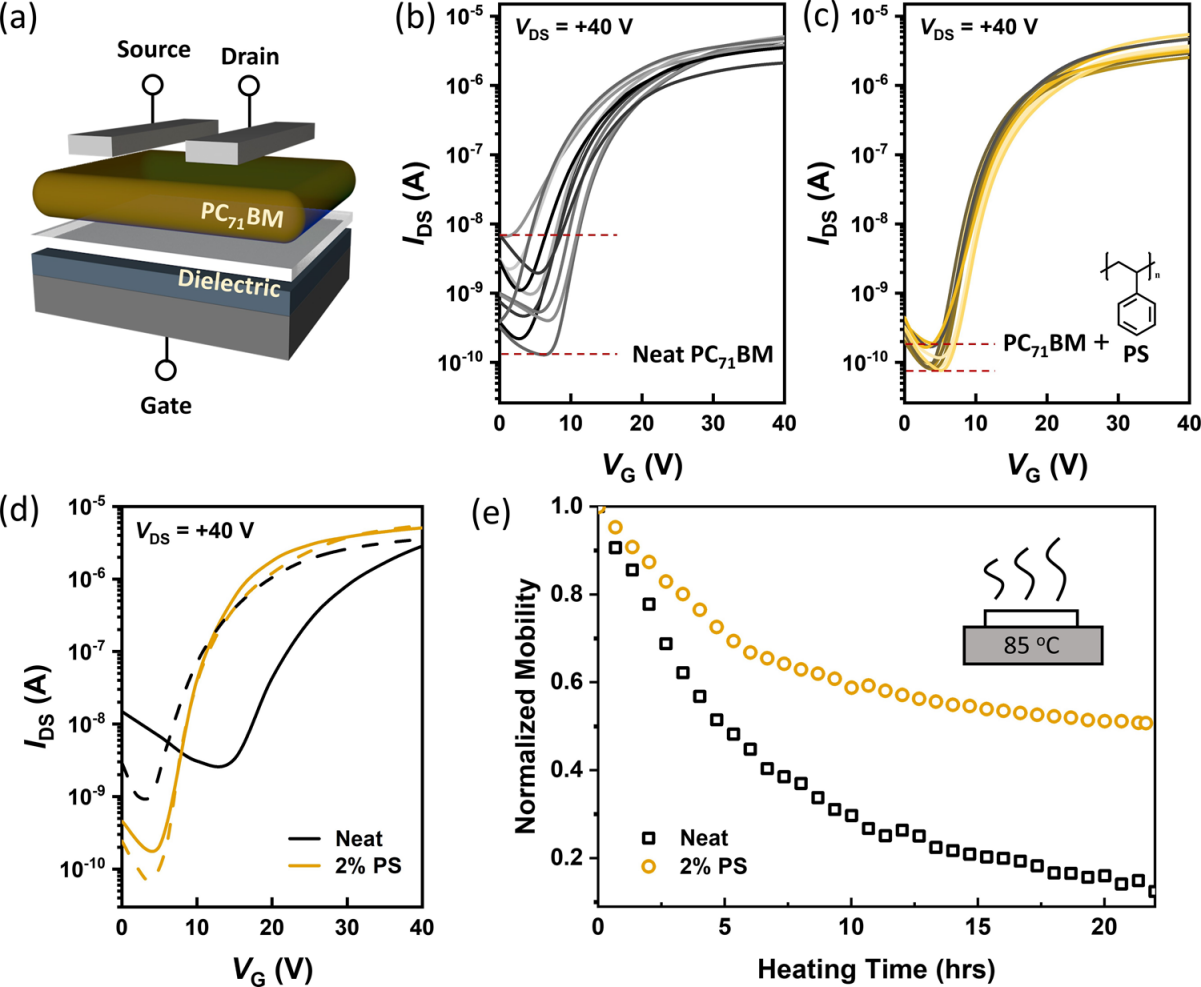
(a) Schematic illustration of OFET configuration. Typical transfer curves of PC_71_BM-based OFETs (b) without and (c) with 2% PS incorporation at room temperature (each curve represents a single batch). (d) Transfer characteristics of neat PC_71_BM and PC_71_BM/PS-based OFETs before (dashed lines) and after heating at 85 ^o^C (solid lines). (e) Normalized field-effect mobility as a function of heating time.

OFETs were first fabricated to investigate the electrical properties of neat PC_71_BM and PC_71_BM/PS (weight ratio of 98:2, MW of PS is 4 kDa) films using a bottom-gate top-contact configuration (Figure 1a). Figure 1b,c shows the typical transfer characteristics of PC_71_BM-based OFETs in different batches, without and with PS incorporation, respectively. Their corresponding output curves and the results of different PS concentrations are shown in Figure S1. When PS is added to PC_71_BM, counterintuitively, the resultant OFETs can still sustain the field-effect mobilities (Table 1), although the electrically inert polymer was expected to impede the charge transport. Two important OFET parameters, *I*_off_ and *SS*, determine the power consumption and the speed of a transistor, closely related to the practical scenarios. However, the neat PC_71_BM devices display relatively high *I*_off_ [(1.2 ± 1.8) × 10^–9^ A] and *SS* (2.8 ± 1.0 V dec^–1^) levels after counting all measured devices (Table 1). Remarkably, 2% PS added in the OFETs can narrow device-to-device parameter spread and effectively reduce *I*_off_ and *SS* to (0.9 ± 0.4) × 10^–10^ A and 1.9 ± 0.2 V dec^–1^, respectively. Evidently, an appropriate amount of PS in the mixture does not impair the electrical performances of PC_71_BM-based OFETs. More interestingly, improved and more consistent OFET parameters (*I*_off_ and *SS*) can be obtained with this facile blending approach. The improvement of *I*_off_ and *SS* levels in the insulator blends was also seen in several prior investigations performed by other researchers.^16,17^ The origin of such improvement has been attributed to the smoothening and depolarization of the underlying gate dielectric layer.^18^

**Table 1:** Summary of OFET Parameters: Field-Effect Mobility, Off Current, Threshold Voltage (*V*_th_), and Subthreshold Swing (*SS*)

sample	mobility (cm^2^ V^−1^ s^−1^)	off current (A)	*V*_th_ (V)	*SS* (V dec^–1^)
neat PC_71_BM	(1.2 ± 0.2) × 10^–2^	(1.2 ± 1.8) × 10^–9^	7.9 ± 2.0	2.8 ± 1.0
PC_71_BM/2% PS	(1.4 ± 0.2) × 10^–2^	(0.9 ± 0.4) × 10^–10^	8.8 ± 0.7	1.9 ± 0.2

#### 2.1.2 Thermal Stability

When the OFETs are heated at a high temperature, e.g., 85 °C, the devices may suffer performance degradation. For example, it has been reported that *I*_off_ in the device increases exponentially with temperature, and the correspondingly increased energy loss is detrimental to the OFETs in practical operating scenarios.^19^ As shown in Figure 1d, for the neat device after heating, *I*_off_ and threshold voltage are shifted up (∼10^–9^ A) and to the right (∼18 V), respectively. Conversely, such a high temperature has no significant effect on OFETs made of blends comprising 2% PS, at which their comparatively low *I*_off_ of ∼10^–10^ A and threshold voltage of ∼8 V contribute to reduced power consumption in the device.

During continuous thermal stress being applied, the OFETs with PS can better maintain their field-effect mobility (Figure 1e) and source-to-drain current (Figure S2). Specifically, the devices retain more than 50% of initial mobility and an on/off ratio of > 5 × 10^4^ after subjecting to heating for 15 h, while the neat devices, in stark contrast, undergo a faster drop in mobility to around 20% of their initial mobility and a smaller on/off ratio of around 10^3^ using the same procedure. It is possibly due to the thermally induced aggregation of PC_71_BM.^20−23^ In addition to thermal stability, the operational stability under continuous bias stress was also studied. Compared with the neat device under high-voltage stress, the PS-added counterpart yields a higher output current (Figure S3a). The transfer curves were measured in a rapid sequence after biasing (Figure S3b). It shows that the neat PC_71_BM-based device exhibits an abnormal transfer shape, while high-voltage stress has a less pronounced impact on the PS-added device. This result further demonstrates that the insulator incorporation can effectively improve the heat dissipation in OSC devices, which can extend their operation lifetime. To verify the generality of heat transfer improvement with the blend approach, we employed the blends of PS with a nonfullerene small-molecule OSC, (2,2′-[[6,6,12,12-tetrakis(4-hexylphenyl)-6,12-dihydrodithieno[2,3-d:2′,3′-d′]-s-indaceno[1,2-b:5,6-b′]dithiophene-2,8-diyl]bis[methylidyne(3-oxo-1H-indene-2,1(3H)-diylidene)]]bis[propanedinitrile]) (ITIC). In ITIC/PS blended devices, similar trends with improved *I*_off_, reproducibility, and thermal stability relative to the neat ITIC devices can be observed in Figure S4.

### 2.2 Heat Transfer

#### 2.2.1 Thermal Diffusivity

**Figure 2:**
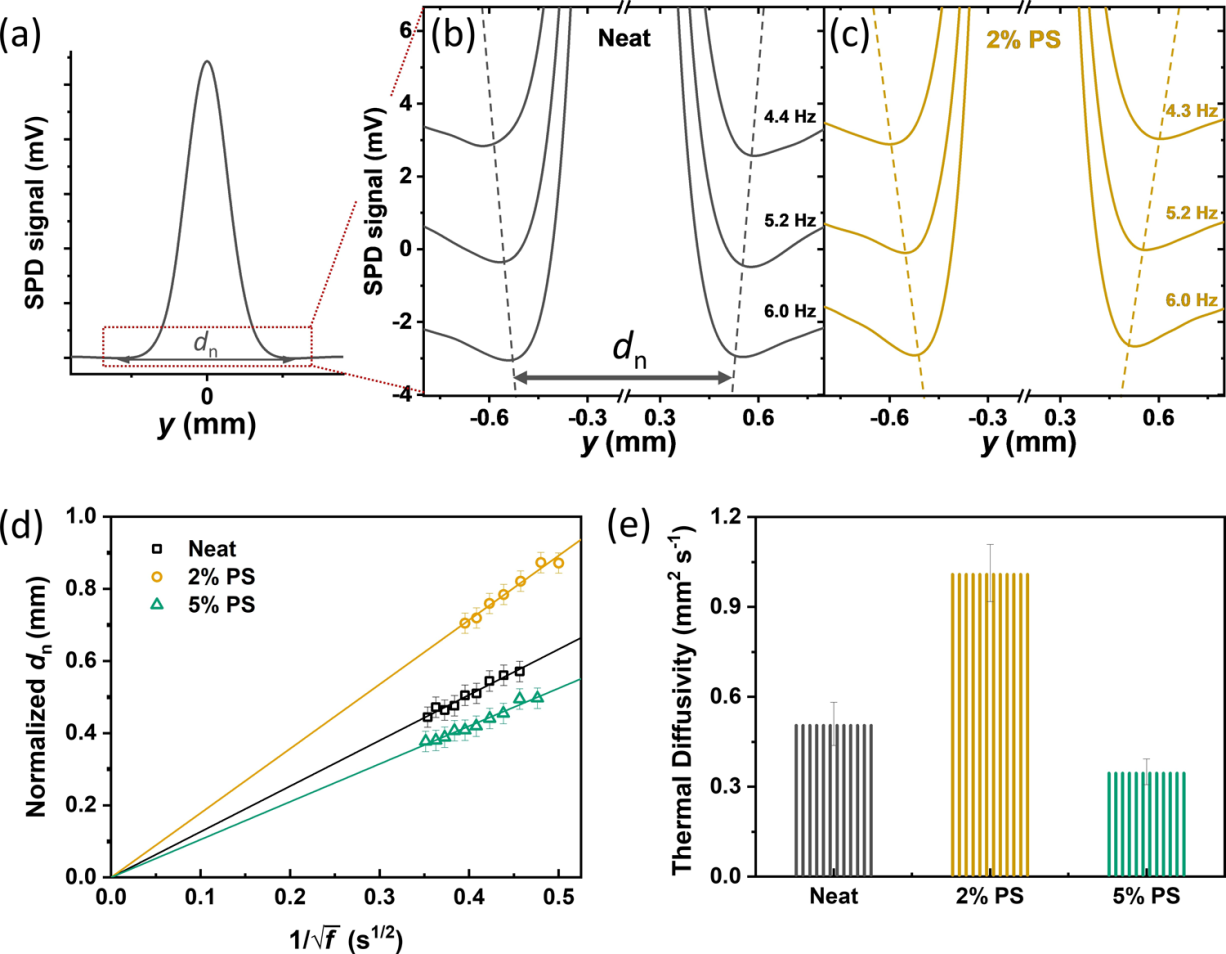
(a) Typical thermal wave pattern measured by the scanning photothermal deflection (SPD). The magnified SPD signals in the red rectangle of (a) at selected modulating frequencies: PC_71_BM films (b) without and (c) with 2% PS addition. (d) Normalized *d_n_* versus 1/√*f* plot. The solid lines are linear fits of eq 1. (e) Thermal diffusivities of PC_71_BM films made using different concentrations of PS.

To explore the origins of the improved thermal stability, we used the highly sensitive scanning photothermal deflection (SPD) technique to probe the thermal diffusivity of thin films without and with PS. Thermal diffusivity quantifies how fast heat propagates or diffuses through the material. The principle of SPD is briefly described below:^24^ the sample is immersed in a transparent deflection fluid and illuminated by a modulated monochromatic pump beam. Due to optical absorption, the sample is heated up and causes a temperature increase in the adjacent deflecting fluid, resulting in its refractive index changes. A laser probe beam launched parallel to and above the sample surface is deflected by changes in the refractive index. The deflection signal can be measured as a function of distance *y* between the pump beam and the probe beam. The resulting SPD signal *versus y* can be interpreted as a surface temperature profile (thermal wave) induced by the heating of the pump beam. Figure 2a shows a typical thermal wave pattern of the lateral temperature profile of the sample upon light illumination, i.e., the SPD signal: The signal reaches a maximum at the center of pump beam irradiation (*y* = 0). When away from the irradiation center, the surface temperature decreases and reaches two minima symmetrically on both sides, with separation *d_n_* between the minima. *d_n_* increases with the decrease of modulated pump frequency (*f*) due to the longer heat diffusion time and the larger spatial temperature gradient. The magnified SPD spectra of measured samples at selected modulating frequencies are shown in Figure 2b,c, and the full SPD spectra can be found in Figure S5. Figure 2d offers the relationship between 1/√*f* and normalized *d_n_* that were deduced from Figure S5. Then, thermal diffusivity *D* can be inferred from the slope of the plot using the following equation^25,26^
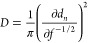
1

The extracted *D* for PC_71_BM films mixed with different concentrations of PS is plotted in Figure 2e. The results show that the thermal diffusivity of the neat PC_71_BM film at room temperature is 0.51 mm^2^ s^–1^ and doubles to 1.01 mm^2^ s^–1^ after adding 2% PS (*D*_PS_ = 0.117 mm^2^ s^–1^),^27^ which is summarized in Table 2. However, the heat diffusion is inhibited when the insulator loading further increases to 5%. The enhancement of thermal diffusivity suggests a better heat transfer ability inside the blended film with an appropriate amount of PS.

**Table 2:** Thermal Diffusivities (*D*) Measured by the SPD Technique^a^

	*D* (mm^2^ s^–1^)	*k* (s^–1^)
neat PC_71_BM	0.51 ± 0.07	0.15
PC_71_BM/2% PS	1.01 ± 0.10	0.27
PC_71_BM/5% PS	0.35 ± 0.04	

a Exponential decay parameters (*k*) fitted to the data (Figure 3C) measured by an infrared thermal camera.

#### 2.2.2 Thermal Images

For a more intuitive demonstration of the difference in heat transfer in the insulator-added films, a cooling test was carried out (Figure 3a). Two samples without and with the insulator were heated at a temperature higher than the surrounding environment and then immediately transferred to the ambient plate (20 °C) to explore how heat is dissipated on the films over time.^28^ The cooling process was monitored by an IR camera, by which the differences in the surface temperature of the samples were visually evident (Figure 3). The surface temperature decays rapidly during the natural cooling process, accompanied by a gradually darkening color. In the beginning, the initial temperature (0 s) of the two samples is identical. After 5 s, the PS-added sample only shows a temperature of around 33 °C, while the control one remains at a higher temperature of about 37 °C (Figure 3b). A similar trend can be observed at the other heating temperatures, with the results shown in Figure S6a. The temperature profiles of each sample can be fitted by Newton’s cooling law with an exponential decay coefficient *k* (Figures 3c and S6b). The extracted *k* for the insulator-containing film (*k* = 0.27 s^–1^) is almost twice that of the neat film (*k* = 0.15 s^–1^) (Table 2). The faster cooling rate in the blended film further supports that the insulator has promoted heat dissipation in OSCs, avoided overheating during operation, and thus improved the device stability. A similar trend with ITIC demonstrates the universality of insulators in improving the thermal behaviors of OSCs (Figure S7).

**Figure 3:**
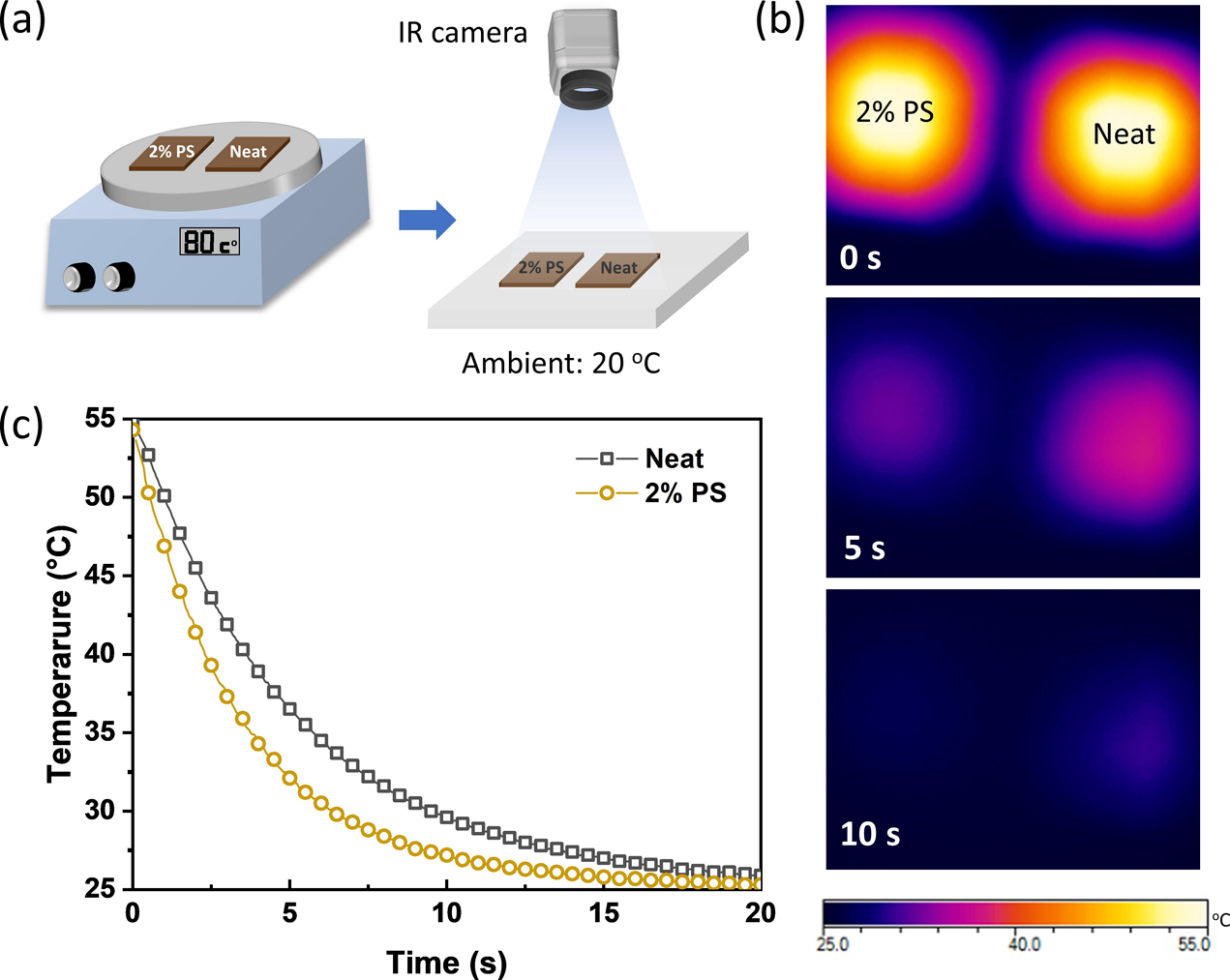
(a) Schematic of the cooling test using an infrared thermal imaging camera (IR camera). (b) Corresponding thermal images at the selected time. (c) Transient variations of surface temperatures based on PC_71_BM films without and with PS incorporation.

### 2.3 PC_71_BM/PS in ETL of Perovskite Solar Cells

**Figure 4:**
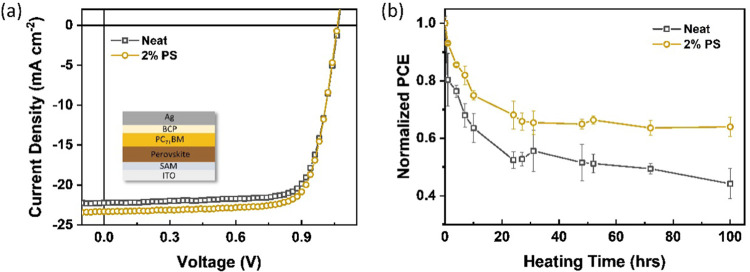
(a) *J*–*V* curves of typical perovskite solar cells (PSCs) without and with PS in the PC_71_BM electron transport layer (ETL). (b) Shelf lifetime tracking of optimized PSCs under constant thermal stress (65 ^o^C) for 100 h.

The previous section offers an observation of the improved heat transfer properties of PC_71_BM/PS blends as a solid film. This observation is general and does not depend on a specific device structure. Therefore, the film can be employed in (i) an n-channel OFET (Section 2.1) or (ii) an electron transport layer (ETL) in other electronic devices. In (ii), we select perovskite solar cells (PSCs), as they are topics of huge concern in thin-film photovoltaics, and our work also provides a new strategy to manage heat dissipation in these photovoltaic cells. Following this line of thought, inverted planar-structured PSCs were fabricated in which fullerene is usually used as an ETL. However, the fullerene ETL has been identified as a key origin of instability in these cells, which has to be addressed promptly, particularly for thermal stability, yet only a few studies have been conducted.^29^ Here, we employ the PC_71_BM/PS film as ETL in PSCs to investigate the effect of PS on the thermal stability of the PSCs. Two beneficial effects were observed. First, the as-prepared cell with the PS-added PC_71_BM film as ETL has a slightly larger short-circuit current (*J*_SC_) and fill factor (FF) and thus an improved average power conversion efficiency (PCE) of 18.34% (Figure 4a), when compared with the PSC in the absence of the insulator (17.87%). Their corresponding characteristic parameters, including PCE, open-circuit voltage (*V*_OC_), *J*_SC_, and FF are summarized in Table S1. Second, the long-term thermal stability of PSCs is improved. As shown in Figure 4b, the measured PCEs degrade monotonically as heating time increases. For the control devices containing the neat PC_71_BM film, only 44% of its initial PCE remains after heating at 65 °C for 100 h. The presence of the insulator in the ETL, in contrast, better stabilizes the PCEs of the devices to resist thermal stress. The PCE can be maintained at nearly 12%, equivalent to ∼64% of its initial PCE under the same test conditions. In summary, in comparison with the neat PC_71_BM film as ETL, photovoltaic parameters can be maintained or even slightly improved when employing the OSC/insulator blends in the PSC field. More importantly, the durability of devices against thermal stress is improved due to the efficient heat transfer in blended films. These results are in line with previous observations, indicating that the insulator-mixed fullerene films can be potentially suitable ETLs for PSCs under high-temperature working conditions.

### 2.4 How PS Improves Thermal Transport?

#### 2.4.1 Morphology

To gain insight into the improved thermal transport in the blended films, the surface morphology of PC_71_BM and ITIC films before and after PS incorporation was investigated by atomic force microscopy (AFM) (Figures S8 and S9). The AFM images reveal that the neat PC_71_BM film possesses a rather flat and featureless surface, consistent with previous reports.^30−32^ The surface morphology remains comparable to that of the neat film upon the addition of 2% PS, whereas the corresponding root-mean-square roughness is reduced from 0.28 to 0.19 nm. The smoother surface suggests that the incorporation of PS aids in the formation of a uniform film.^33^ However, when the PS concentration is increased to 5%, distinct phase separation can be observed (Figure S8), which may explain the suppressed thermal diffusivity of PC_71_BM at high PS loading (Figure 2e). The probed X-ray diffraction (XRD) spectra show that incorporating 2% PS does not exert a significantly observable effect on the crystallinity of the PC_71_BM film, as shown in Figure S10a. Together with similar absorption spectra of the neat and PS-added films (Figure S10c), it is noticeable that the surface morphology and crystalline packing of OSC films are quite insensitive to 2% PS inclusion.

#### 2.4.2 Chain Length Effect

It has been reported that the molecular weight (MW) (or the chain length) of the insulating polymer has a substantial impact on the device performances of OSC/insulator blends.^17,34,35^ To investigate the MW effect, we used another high-MW PS, 1000 kDa PS, to fabricate blended OFETs, as shown in Figure S11a. Unlike previous blends with lower MW 4 kDa of PS, the *I*_off_ of the devices blended with 1000 kDa PS undergoes an upward shift, even higher than the neat PC_71_BM device. In addition to the inferior OFET performance, the high-MW-based blends yield slower heat dissipation when directly compared with the neat film (Figure S11b). These findings indicate that the electrical and thermal behaviors of the blends strongly depend on the MW of PS.

**Figure 5:**
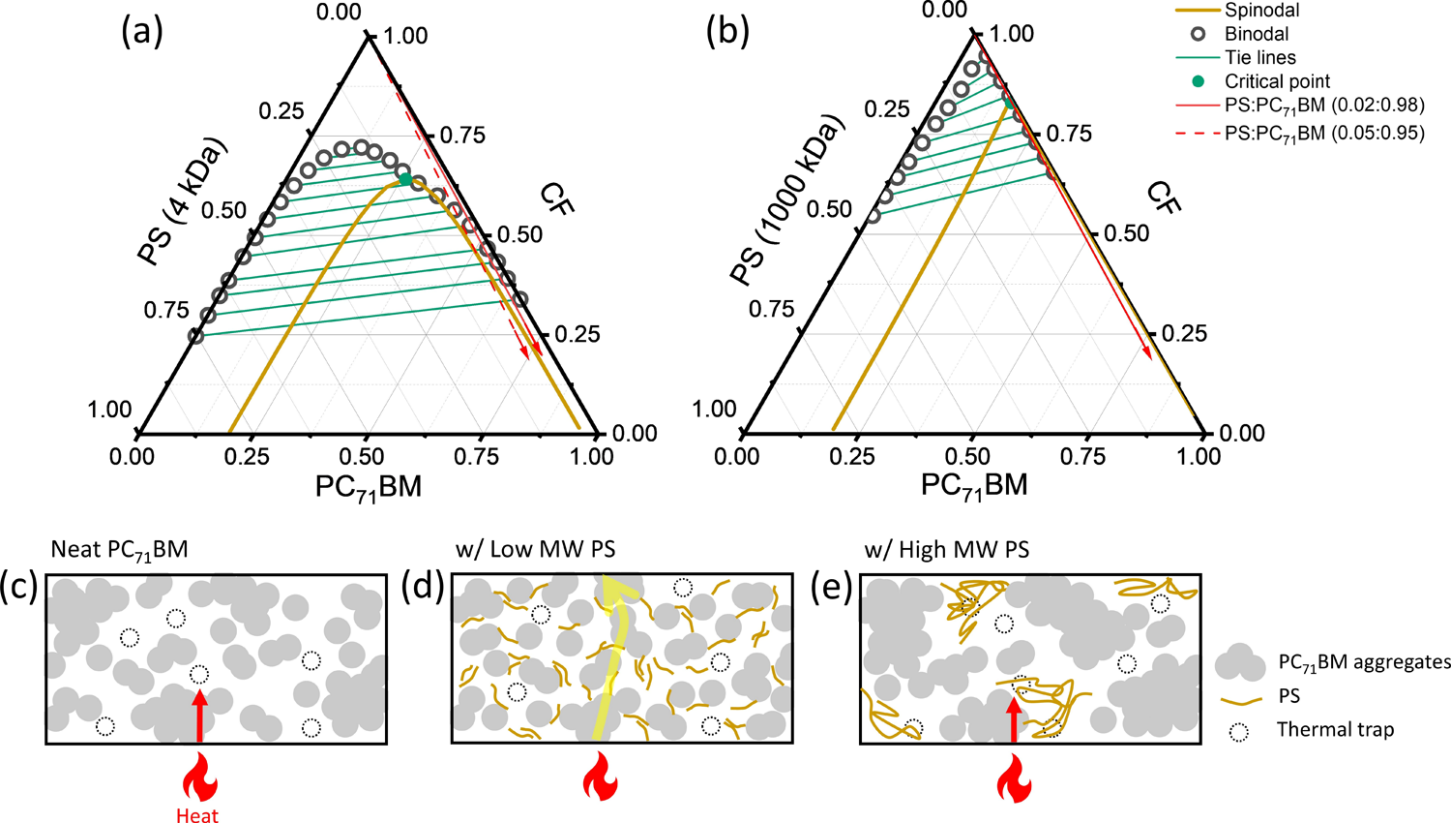
Phase diagrams for the ternary PC_71_BM/PS/chloroform (CF) system with two different molecular weights (MWs) of PS: (a) MW = 4 kDa and (b) MW = 1000 kDa. The intersection of binodal and spinodal lines is the critical point. The red arrows denote the solvent quenching line with PC_71_BM:PS weight ratios of 98:2 (solid arrow) and 95:5 (dashed arrow). (c–e) Schematic illustration of neat OSC and blended films made using two different MWs of PS.

To further explore the underlying mechanism and understand the phase behaviors of components in the blended system, the ternary phase diagrams consisting of PS, PC_71_BM, and solvent components were calculated based on Flory–Huggins theory, as shown in Figure 5a,b (the calculation parameters can be found in Tables S2 and S3 and the details are discussed in the Supporting Information). The single-phase and phase-separated regions are distinguished by the binodal line, i.e., the miscibility boundary, which is determined by the chemical potential equilibrium of liquid phases. The spinodal line and critical point are derived from the second and third derivatives of Gibbs free energy, respectively. Spinodal decomposition takes place in the region below the spinodal, where liquid–liquid (L–L) phase separation occurs spontaneously due to the repulsive interaction between PS and PC_71_BM molecules. Tie lines represented by green lines connect the binodal compositions with equal chemical potentials.

Figure 5a,b shows that adding a certain amount of PS with different MWs has significantly diverse effects on the ternary phase diagram. In the low-MW-based system (Figure 5a), a small miscibility gap, denoted as open dots, indicates a low probability of presolidification phase separation and favorable miscibility.^36^ In the presence of a high-MW polymer (Figure 5b), the miscibility gap expands and almost covers the entire diagram. The asymmetry of binodal is due to the large size difference between PC_71_BM and high-MW polymer.^37^ Obviously, the ternary system composed of high-MW PS is less miscible than the one containing low-MW PS. The red arrows in Figure 5a,b are the solvent quenching lines that indicate the changes in the overall composition of the mixed solution upon solvent evaporation,^38^ in which the solid arrows represent the subsequent continuous evaporation of the PC_71_BM:PS mixture with a weight ratio of 98:2. Upon further increasing PS concentration to 5%, the solvent quenching line (red dashed arrow) in the ternary phase diagram will directly enter the thermodynamically unstable region below the spinodal (yellow line), where L–L demixing is present, as already hinted by AFM results (Figure S8d). The same goes for the solvent quenching line in the case of high-MW-based blends with a PC_71_BM:PS weight ratio of 98:2 (Figure 5b), in which solvent evaporation drives the system into the unstable part of the phase diagram, triggering the separation of PC_71_BM and high-MW PS phases.

Based on the phase analysis, Figure 5c–e offers a model to explain the effect of the PS chain length on the heat transfer of blends. In the neat PC_71_BM film (Figure 5c), the predominantly isolated PC_71_BM domains are formed due to the highly localized vibrations, resulting in poor heat conduction.^12^ In addition, the fullerene aggregation under thermal stress worsens the situation. When low-MW PS is added to PC_71_BM, the intimacy of mixing demonstrated by a small miscibility gap means that more low-MW polymers are prone to distribute in the PC_71_BM domains, hindering the growth of PC_71_BM aggregates, consequently allowing the formation of interconnected phonon transport network, in which PS can bridge effective conductive regions providing “highways” for phonon propagation due to the efficient phonon transport of PS chains (Figure 5d).^39,40^ When the high-MW PS is blended with PC_71_BM, the mixtures tend to separate into individual phases due to the repulsive interaction between OSC and PS. In this two-phase structure, PS phases are too “pure” and scatter phonons as impurities, disrupting the percolation pathways, thus hampering the phonon transport in the blended film (Figure 5e). Therefore, PS needs to be well mixed with OSC and avoid two-phase demixing to achieve efficient phonon propagation in the blends.

## 3 Conclusions

This work studies the effect of PS on the thermal performances of OSCs, even though PS is generally considered to be electrically and thermally insulating. The addition of PS can effectively boost the heat transfer of OSCs, which can be reflected by the improved thermal stability in OFET and PSC configurations, the increased thermal diffusivity in the SPD technique, and the enhanced heat dissipation indicated by an IR camera. These improvements are achieved without sacrificing electrical properties. The computed ternary phase diagrams suggest that the low-MW PS is intimately mixed with OSC, which enables efficient phonon transport by forming percolated PC_71_BM/PS phonon pathways. In contrast, the separation between the high-MW insulator and OSC phases in the blends leads to poor heat conduction. The insulator blend approach provides a facile path for diverse thermal management and thermal-based applications by tuning the thermal properties of OSCs.

## 4 Experimental Section

### 4.1 Materials and Thin-Film Fabrication

PC_71_BM, PS, and ITIC were purchased from Nano-C, Polymer Source, and 1-Material Inc., respectively. All of the materials were used as received without further purification. The OSC solution has a concentration of 10 mg mL^–1^ in anhydrous chloroform (Sigma Aldrich). PS with the same concentration was separately prepared and stirred in a glovebox overnight before using. Then, the PS solution was added to the OSC solution directly at various compositions to form different blended OSC/PS solutions. The active layer was spin-coated at a speed of 1400 rpm for 30 s using these solutions in a nitrogen-filled glovebox.

### 4.2 Device Fabrication

#### 4.2.1 OFET Fabrication

The Si/SiO_2_ wafers were subjected to ultrasonication and rinsed with deionized water, acetone, and 2-propanol for 20 min each, followed by drying with compressed air. After being treated with an ultraviolet–ozone cleaner (Jelight, UVO cleaner, Model 42–220) for 13 min, the substrates were transferred to a glovebox for spin coating. Poly(2,3,4,5,6-pentafluorostyrene) (PPFS) dissolved in methyl isobutyl ketone with a concentration of 10 mg/mL was spin-coated at 2000 rpm for 60 s as the gate dielectric layer.^41^ The active layers were spin-coated on the top of the PPFS film without further annealing. Finally, LiF/Al (1 nm/100 nm) electrodes were coated on the samples in a high vacuum chamber (∼4 × 10^–6^ Torr) through a shadow mask, defining a 50 μm channel length.

#### 4.2.2 PSC Fabrication

The Indium tin oxide (ITO) patterned substrates were cleaned prior to the device fabrication by sonication in deionized water, acetone, and 2-propanol, respectively. The self-assembled monolayer molecule ([2-(3,6-dimethoxy-9H-carbazol-9-yl)ethyl]phosphonic acid) (MeO-2PACz) was spin-coated on the substrates. Then, the perovskite film (CH_3_NH_3_PbI_3_) was spin-coated with the typical one-step solution method.^42^ After that, PC_71_BM or PC_71_BM/PS film and bathocuproine (BCP) film were deposited on the active layer, respectively. Finally, 80 nm of Ag was evaporated on the top of films and served as the cathodes in a high vacuum chamber.

### 4.3 Characterization

All OFET measurements were taken in a cryostat (Oxford Instruments, Optistat DN-V) under vacuum (below 10^–4^ Torr) and dark conditions. During OFET measurements, a Keithley 236 source measurement unit was used to provide the source-to-drain bias, while a Xantrex XT 120-0.5 was used to supply the gate voltage. The field-effect mobilities were extracted from the saturation regime of transfer curves. In SPD measurement, the samples were immersed into perfluorohexane as the deflection fluid and irradiated by a 5 mW, 532 nm laser diode as the pump beam. The pump beam was modulated by a mechanical chopper with specific frequencies and focused by a convex lens. The deflection of a probe beam (2 mW 632 nm He–Ne laser) on the sample surface due to the released heat was detected by a silicon PIN photoquadrant detector (TEMic). A position sensor, together with a chopper, was connected to the Standford Research SR830 lock-in amplifier to collect the data. The thermal images were taken by an IR camera (Optris PI 400i). The surface morphology of the thin films was probed by an atomic force microscope (AFM) (Veeco Deltak 150 surface profiler) operating in tapping mode.

## Supporting Information Available

OFET output characteristics, thermal and bias stress test, OFET characteristics of the ITIC system, SPD signals, transient variations of surface temperature, photovoltaic parameters of perovskite solar cells, AFM topography, XRD spectra, optical absorption spectra, and computational details about the ternary phase diagrams (PDF)
