# Supplementary material for: Heat Transfer Enhancement of n-Type Organic Semiconductors by an Insulator Blend Approach
Source: ACS Appl Mater Interfaces. 2022 Jun 23;14(26):30174–81. doi: 10.1021/acsami.2c05503 (PMC9264312; doi:10.1021/acsami.2c05503)
Supplement: Supplementary file 1 — am2c05503_si_001.pdf [file am2c05503_si_001.pdf]

Supporting Information

Heat transfer enhancement of n-type organic semiconductors by an insulator blend approach

Zhuoqiong Zhang[a,b], Yabing Tang[c], Yunfan Wang[d], Zixin Zeng[d], Run Shi[b], Han Yan[c], Sai-Wing Tsang[d], Chun Cheng[b]* and Shu Kong So[a]*

[a]Department of Physics and Institute of Advanced Materials, Hong Kong Baptist University, Kowloon Tong, Hong Kong SAR 999077, P. R. China

[b]Department of Materials Science and Engineering, Southern University of Science and Technology, Shenzhen 518055, P. R. China

[c]State Key Laboratory for Mechanical Behavior of Materials, Xi'an Jiaotong University, Xi'an 710049, P. R. China

[d]Department of Materials Science and Engineering, City University of Hong Kong, Hong Kong SAR 999077, P. R. China

*Correspondence: S. K. S. (email: skso@hkbu.edu.hk); C. C. (email: chengc@sustech.edu.cn)

1. OFET characteristics

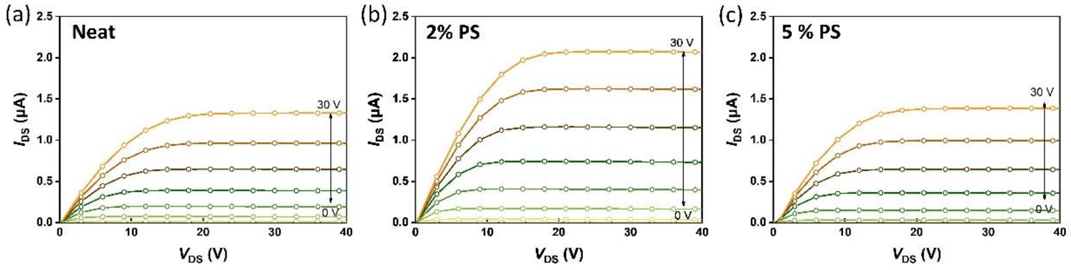

Figure S1 Output characteristics of $PC_{71}BM$/PS based OFETs with (a) 0%, (b) 2%, and (c) 5% PS content, respectively.

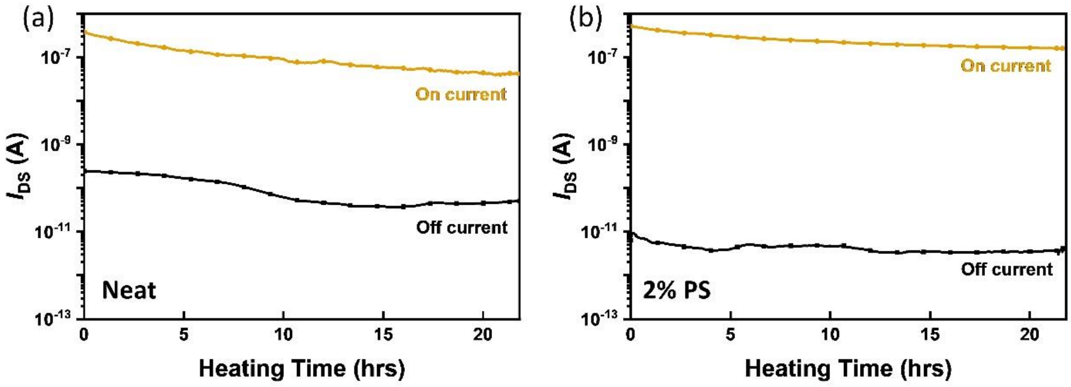

Figure S2 Time-dependent off and on current of $PC_{71}BM$ based OFETs (a) without and (b) with PS incorporation under 85 ºC thermal stress (V_{DS} = +40 V).

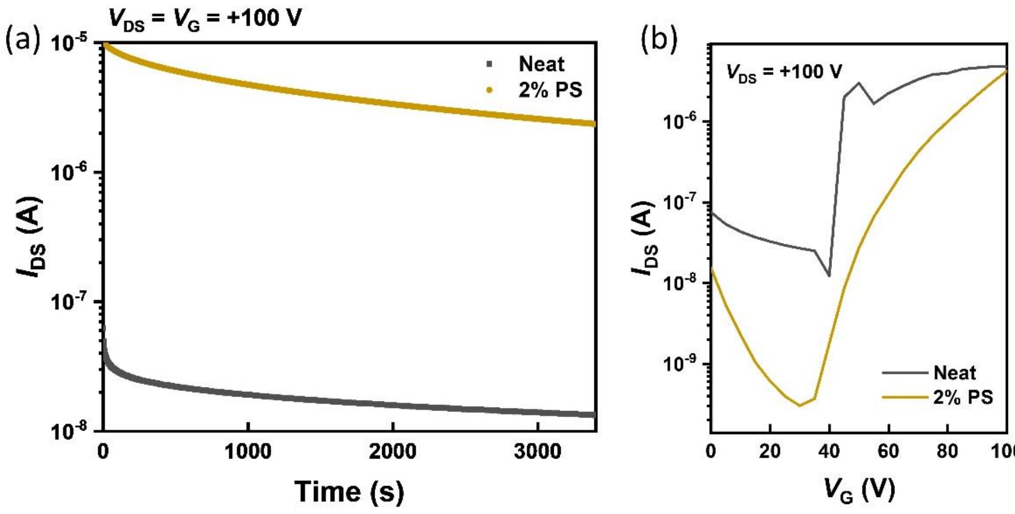

Figure S3 (a) High-voltage bias stress for $PC_{71}BM$ based OFETs without and with PS incorporation. (b) Their corresponding transfer curves after bias stress.

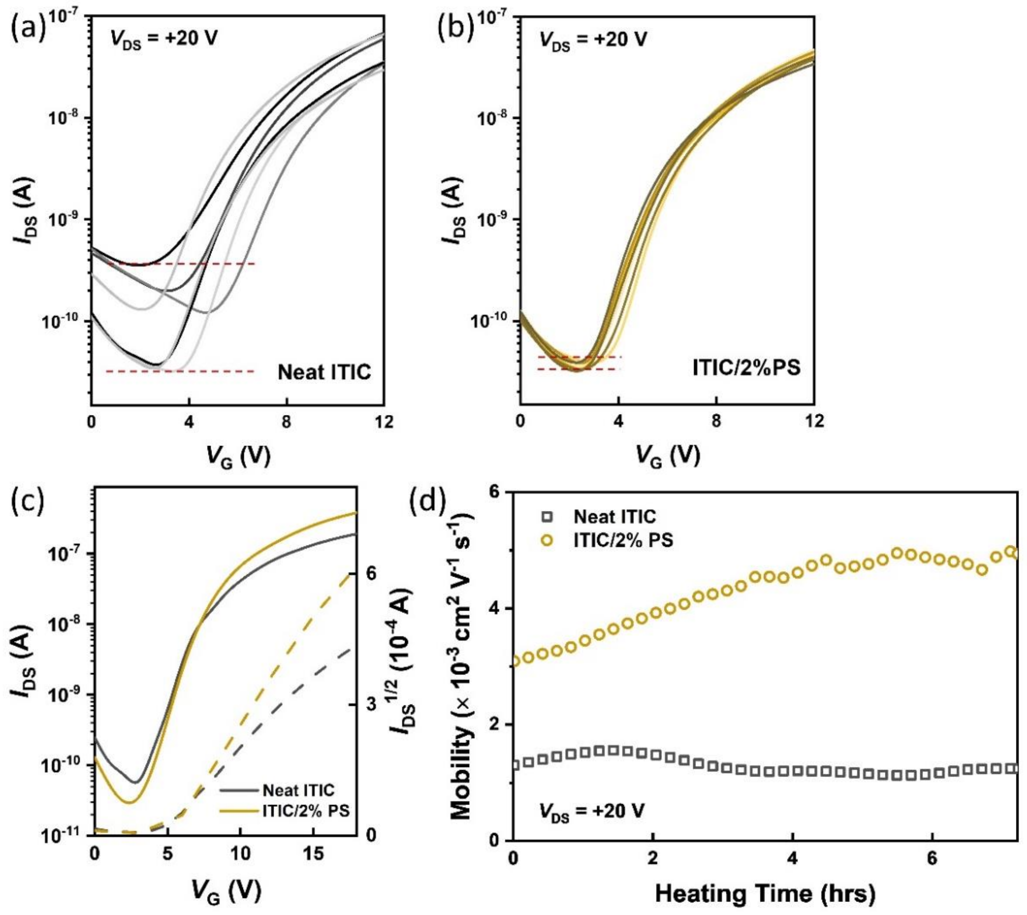

Figure S4 Transfer curves of ITIC-based OFETs (a) without and (b) with PS incorporation at room temperature. (c) The corresponding transfer curves at 85 ºC. (d) Field-effect mobilities at 85 ºC as a function of heating time.

2 Heat transfer

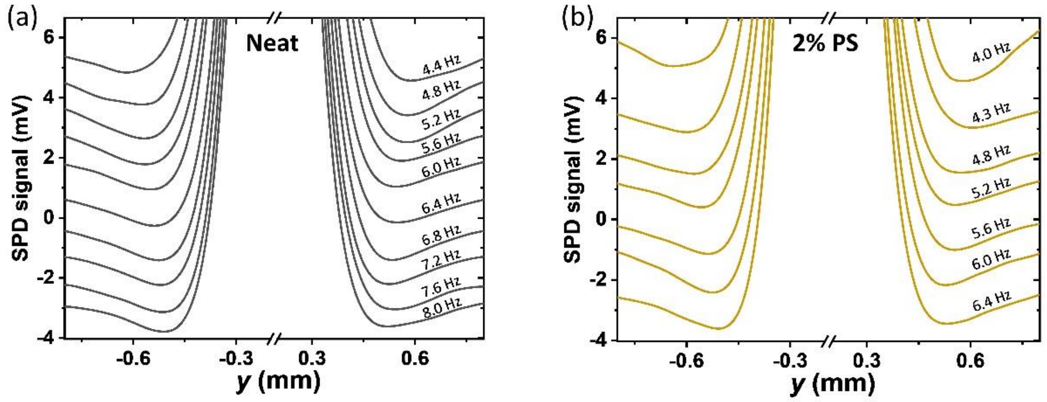

Figure S5 SPD signals for (a) $PC_{71}BM$ and (b) $PC_{71}BM$/2% PS films at different modulation frequencies.

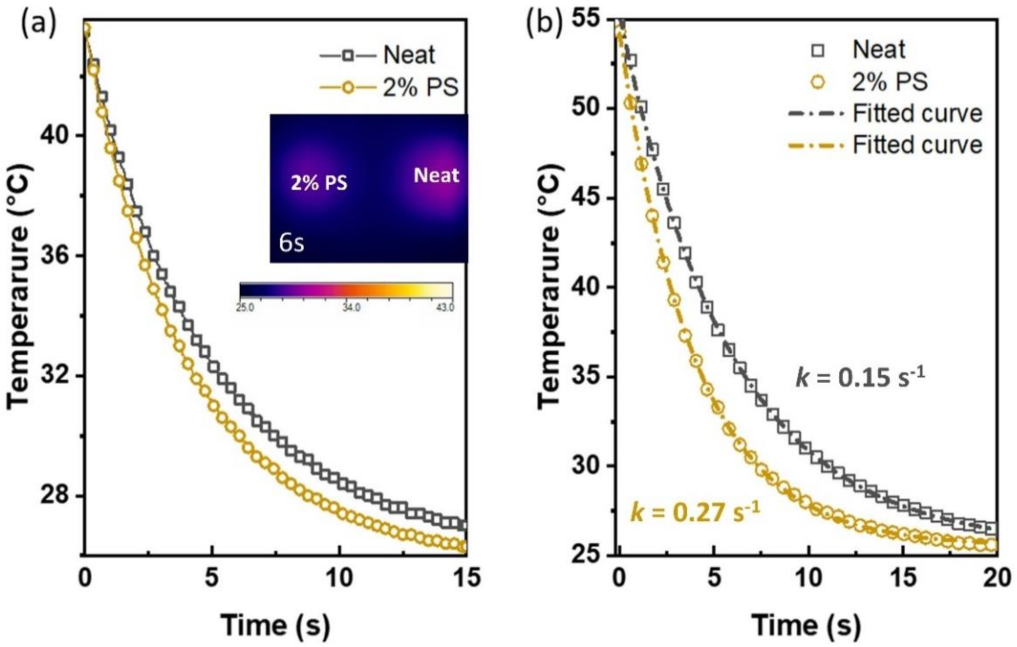

Figure S6 (a) Transient variations of surface temperatures based on $PC_{71}BM$ films without and with PS incorporation (inset: corresponding thermal images at 6s). Neat $PC_{71}BM$ film shows little temperature decline in comparison with $PC_{71}BM$/PS one. The overall slowed-down heat transfer is due to the low thermal gradient. (b) The experimental temperature profiles (open symbols) are fitted by Newton's cooling law ($T \propto e^{-kt}$, T is the temperature, k is the exponential decay coefficient and t is the time).

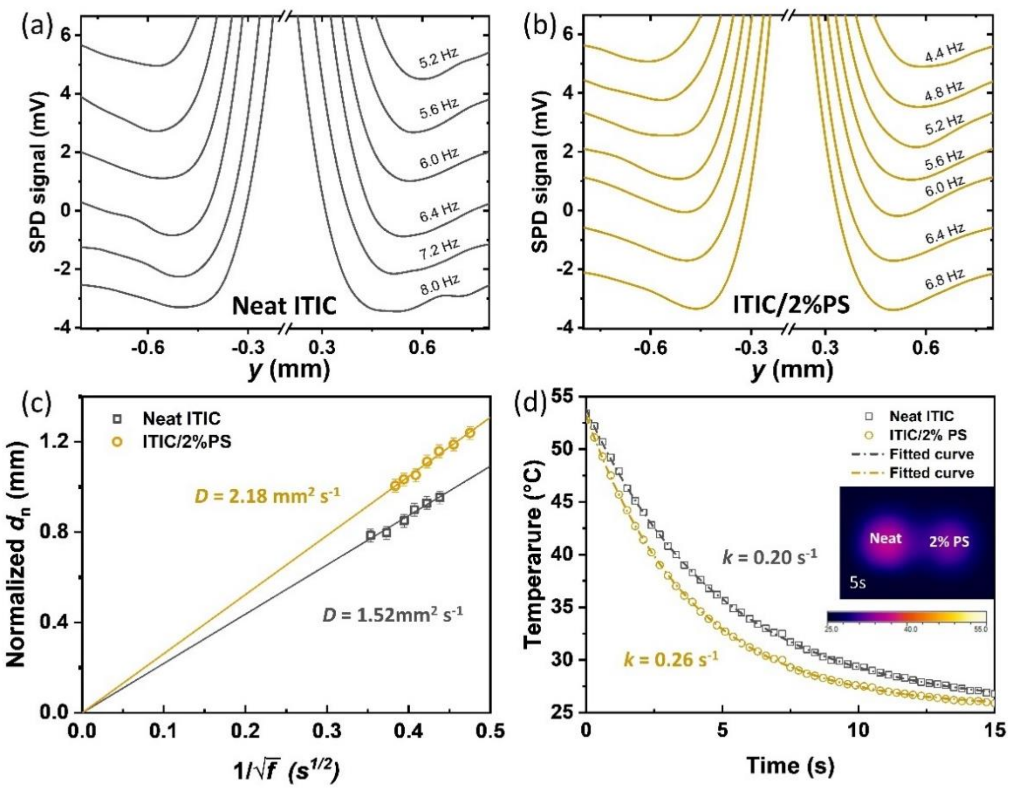

Figure S7 SPD signals for (a) ITIC and (b) ITIC/2% PS films at different modulation frequencies. (c) Corresponding normalized d_n versus $1/\sqrt{f}$ plot. D is the thermal diffusivity value. (d) Transient variations of surface temperatures (inset: corresponding thermal images at 5s) based on ITIC films without and with PS incorporation.

3. $PC_{71}BM$/PS in ETL of perovskite solar cell

Table S1 Photovoltaic parameters (average of five individual cells) of perovskite solar cells based on different ETLs.

ETL	PCE (%)	PCE_{max} (%)	V_{OC} (V)	J_{SC} (mA cm^{-2})	FF (%)
Neat $PC_{71}BM$	17.87 ± 0.45	18.57	1.07 ± 0.01	22.42 ± 0.44	74.38 ± 1.49
$PC_{71}BM$/2% PS	18.34 ± 0.36	18.85	1.06 ± 0.01	22.90 ± 0.28	75.20 ± 0.96

4. How PS improves thermal transport?

4.1 Morphology

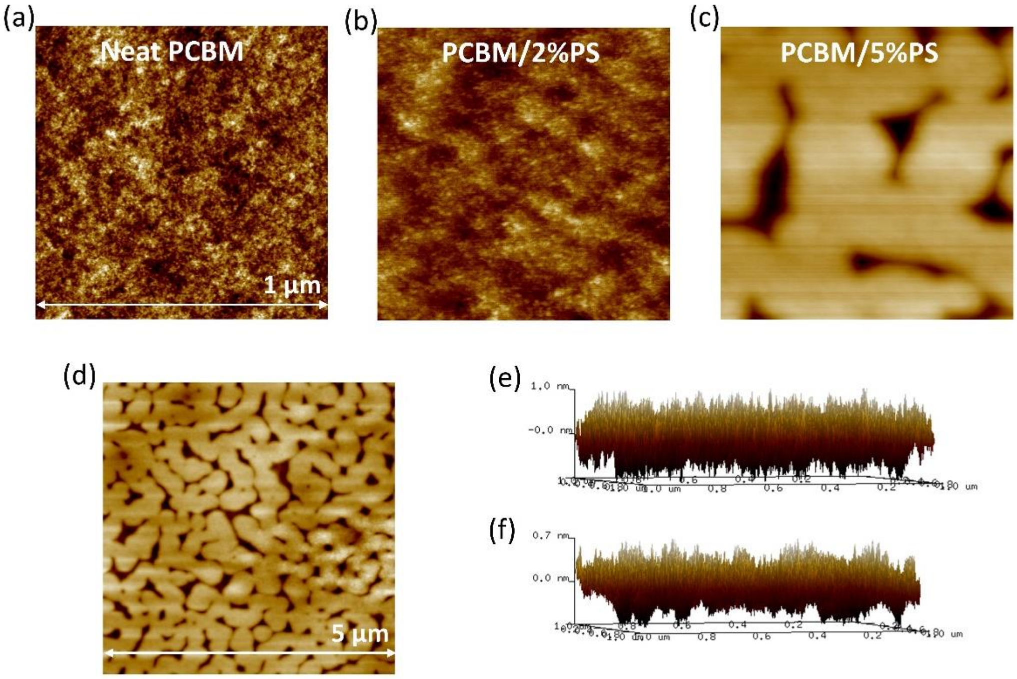

Figure S8 (a–c) Height AFM topography images recorded in tapping mode for $PC_{71}BM$ films made with different PS concentrations. The size of each scan is 1 µm × µm. (d) The magnified AFM topographic image of $PC_{71}BM$/5% PS based film. The size is 5 µm × µm. (e,f) Corresponding height images of panels (a) and (b), respectively.

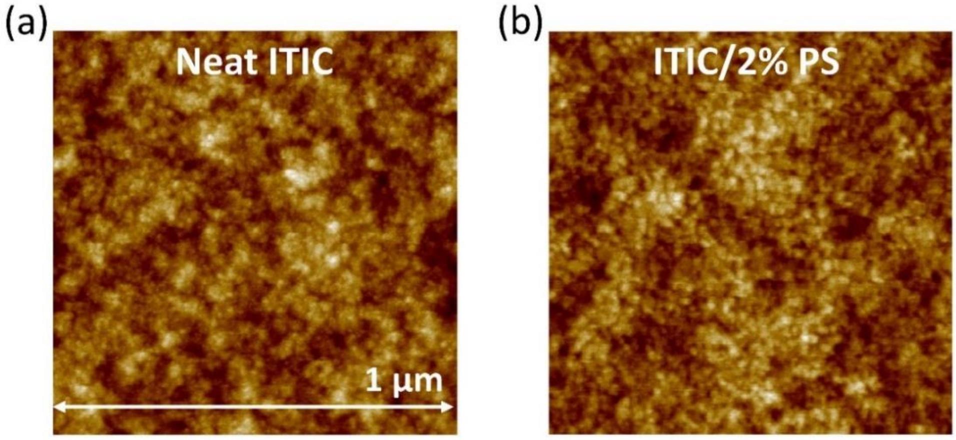

Figure S9 AFM topography images recorded in tapping mode for ITIC films (a) without and (b) with PS incorporation. The size of each scan is 1 μm × μm.

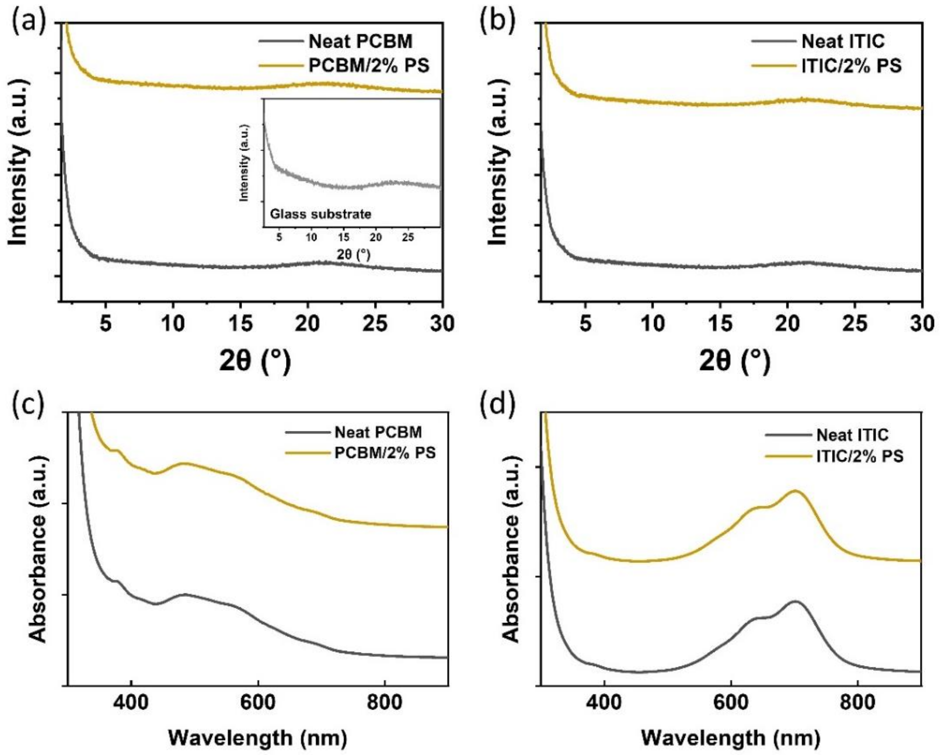

Figure S10 X-ray diffraction (XRD) spectra of (a) $PC_{71}BM$ and (b) ITIC films without and with PS incorporation. (Inset of a: corresponding XRD spectra of glass substrate) Optical absorption spectra of (c) $PC_{71}BM$ and (d) ITIC films without and with PS incorporation. XRD and absorption results based on neat $PC_{71}BM$ film are consistent with the previous report.[1,2]

4.2 Chain length effect

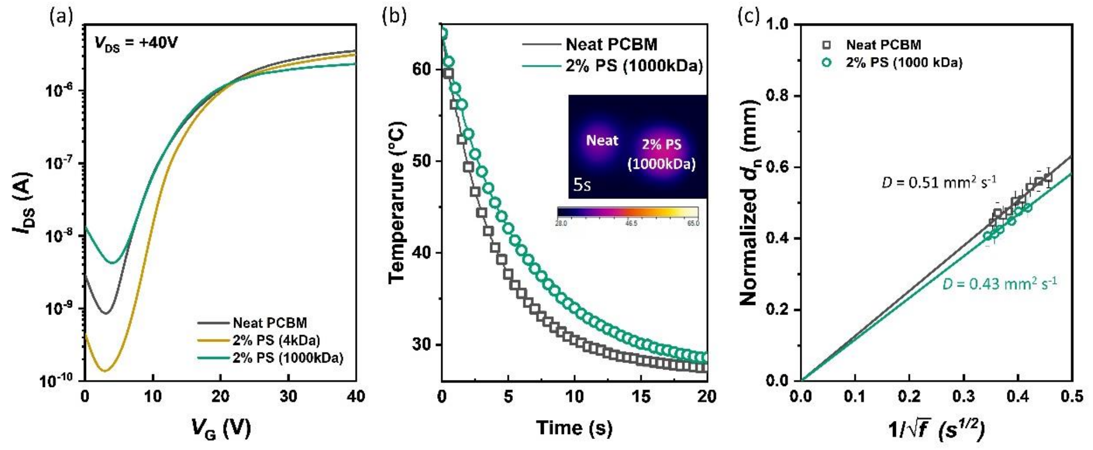

Figure S11 (a) Transfer curves of neat $PC_{71}BM$ and $PC_{71}BM$/2% PS OFETs made with two different molecular weight (MW) of PS. (b) Transient variations of surface temperatures (inset: corresponding thermal images at 5s) based on $PC_{71}BM$ films without and with PS (MW = 1000 kDa) incorporation. (c) Corresponding normalized d_n versus $1/\sqrt{f}$ plot obtained by SPD technique. D is the thermal diffusivity value.

The ternary phase diagram:

The Flory–Huggins theory assumes the free energy of mixing comes from the molecular size and interaction, i.e., the entropic and enthalpic contributions. The polymer chain topology or segmental packing will not be considered. Note that the polymers in this study are monodisperse. The Gibbs free energy of mixing (ΔG) for a ternary system is given by (subscripts 1, 2, and 3 respectively refer to solvent, PS, and $PC_{71}BM$):[3,4]

$$\frac{\Delta G}{RT} = n_1 \ln \varphi_1 + n_2 \ln \varphi_2 + n_3 \ln \varphi_3 + \chi_{12}\, n_1\, \varphi_2 + \chi_{13}\, n_1\, \varphi_3 + \chi_{23} N_2\, n_2\, \varphi_3 \quad (1)$$

where R is the universal gas constant, T is the temperature, n_i is the number of moles, φ_i is the volume fraction ($\sum_{i=1}^{3} \varphi_i = 1$ in the incompressibility assumption), N_i is the

site volume (relative degrees of polymerization), and χ_{ij} is the interaction parameters between mixed molecules (here it is assumed a negligible effect of ternary interaction parameter $\chi_{123} = 0$). Furthermore, χ can be evaluated by Hildebrand solubility parameters (δ) using the following expression:[5,6]

$$\chi_{ij} = \frac{V_m}{RT}(\delta_i - \delta_j)^2 + \chi_s \quad (2)$$

where V_m is the monomeric molar volume of solvent, χ_s represents the entropic contribution to the interaction parameter and typically adopts an empirical value of 0.34.[6,7] For apolar materials, δ is proportional to the square root of surface energy (γ) with the following relationship:[5,6]

$$\delta = K\sqrt{\gamma} \quad (3)$$

where K is a proportionality constant equals 116×10^3 $m^{-1/2}$.[5,6] The corresponding parameters are extracted from literature for a reasonable range, some of which are given in Table S3. According to different reports, it can be noted that PS has a wide range of surface energy.[7,8] We adopted the value from ref. 9–11 because χ_{12} obtained from this value is more consistent with the previous reports.[12,13] Here, the surface energy of PS is assumed to be a constant with different molecular weights (MWs) due to its weak dependence on MW.[7,14] We have verified that the actual value of PS surface energy does not affect the outcome of modeling in a significant way.

The binodal line is determined by the chemical potential equilibrium of liquid phases and obtained by an iterative process, in which the chemical potential $\Delta\mu_i$ can be expressed as: $\Delta\mu_i = \frac{\partial \Delta G}{\partial n_i}$. The binodal can be calculated based on the equations below:[3]

$$\frac{\Delta\mu_1}{RT} = \ln\varphi_1 + (1-\varphi_1) - \frac{\varphi_2}{N_2} - \frac{\varphi_3}{N_3} + (\chi_{12}\varphi_2 + \chi_{13}\varphi_3)(\varphi_2 + \varphi_3) - \chi_{23}\varphi_2\varphi_3 \quad (4)$$

$$\frac{\Delta\mu_2}{RT} = \ln\varphi_2 + (1-\varphi_2) - \varphi_1 N_2 - \frac{\varphi_3 N_2}{N_3} + (\chi_{12}\varphi_1 N_2 + \chi_{23} N_2 \varphi_3)(\varphi_1 + \varphi_3) - \chi_{13}\varphi_1\varphi_3 N_2 \quad (5)$$

$$\frac{\Delta\mu_3}{RT} = \ln\varphi_3 + (1-\varphi_3) - \varphi_1 N_3 - \frac{\varphi_2 N_3}{N_2} + (\chi_{13}\varphi_1 N_3 + \chi_{23} N_3 \varphi_2)(\varphi_1 + \varphi_2) - \chi_{12}\varphi_1\varphi_2 N_3 \quad (6)$$

$$\Delta\mu_{i,A} = \Delta\mu_{i,B} \quad (7)$$

$$\varphi_{1,A} + \varphi_{2,A} + \varphi_{3,A} = \varphi_{1,B} + \varphi_{2,B} + \varphi_{3,B} = 1 \quad (8)$$

The spinodal, i.e., the limit of stability, can be calculated based on the equation below:[3,5]

$$\frac{\partial^2\Delta G}{\partial\varphi_2{}^2}\frac{\partial^2\Delta G}{\partial\varphi_3{}^2} - \frac{\partial^2\Delta G}{\partial\varphi_2\varphi_3}\frac{\partial^2\Delta G}{\partial\varphi_2\varphi_3} = 0 \quad (9)$$

where the second order derivatives of Gibbs free energy are described by:[3,5]

$$\frac{\partial^2(\frac{\Delta G}{RTn_{totol}})}{\partial\varphi_2{}^2} = \frac{1}{\varphi_1} + \frac{1}{\varphi_2 N_2} - 2\chi_{12} \quad (10)$$

$$\frac{\partial^2(\frac{\Delta G}{RTn_{totol}})}{\partial\varphi_3{}^2} = \frac{1}{\varphi_1} + \frac{1}{\varphi_3 N_3} - 2\chi_{13} \quad (11)$$

$$\frac{\partial^2(\frac{\Delta G}{RTn_{totol}})}{\partial\varphi_2\varphi_3} = \frac{1}{\varphi_1} + \chi_{23} - \chi_{13} - \chi_{12} \quad (12)$$

Table S2 Parameters of molar volume (V_m), molecular weight of repeating unit (M_{wt}), surface energy (γ) and solubility parameter (δ) for various PS, $PC_{71}BM$ and solvent.

**Materials**	V_m (cm^3 mol^{-1})	M_{wt} (g mol^{-1})	γ (mN m^{-1})	δ ($MPa^{1/2}$)
$PC_{71}BM$	607.3[ref.15]	910.88	35.8[ref.5]	22.0
PS (4 kDa)	99.2[ref.16,17]	104.1	32.4	20.9
PS (1000 kDa)	99.2[ref.16,17]	104.1	32.4	20.9
Chloroform	80.2	119.38		18.9

Table S3 Flory–Huggins interaction parameters.

χ	CF	PS (4 kDa)	PS (1000 kDa)	$PC_{71}BM$
CF	0	0.47	0.47	0.65
PS (4 kDa)	0.47	0	–	0.38
PS (1000 kDa)	0.47	–	0	0.38
$PC_{71}BM$	0.65	0.38	0.38	0

Reference:

1 A. Viterisi, F. Gispert-Guirado, J. W. Ryan and E. Palomares, *J. Mater. Chem.*, 2012, **22**, 15175.
2 Q. Zhang, C. Bao, S. Cui, P. Zhong, K. Zhang, W. Zhu and Y. Liu, *J. Mater. Chem. C*, 2020, **8**, 16551–16560.
3 Y. Tang, B. Lin, H. Zhao, T. Li, W. Ma and H. Yan, *ACS Appl. Mater. Interfaces*, 2020, **12**, 13021–13028.
4 F. A. Bokel, S. Engmann, A. A. Herzing, B. A. Collins, H. W. Ro, D. M. DeLongchamp, L. J. Richter, E. Schaible and A. Hexemer, *Chem. Mater.*, 2017, **29**, 2283–2293.
5 J.-H. Kim, A. Gadisa, C. Schaefer, H. Yao, B. R. Gautam, N. Balar, M. Ghasemi, I. Constantinou, F. So, B. T. O'Connor, K. Gundogdu, J. Hou and H. Ade, *J. Mater. Chem. A*, 2017, **5**, 13176–13188.
6 S. Kouijzer, J. J. Michels, M. van den Berg, V. S. Gevaerts, M. Turbiez, M. M. Wienk and R. A. J. Janssen, *J. Am. Chem. Soc.*, 2013, **135**, 12057–12067.
7 A. Kunz, P. W. M. Blom and J. J. Michels, *J. Mater. Chem. C*, 2017, **5**, 3042–3048.

8 R. E. Smith, Surface Energy Data for PS: Polystyrene, CAS #9003-53-6, http://www.accudynetest.com/polymer_surface_data/polystyrene.pdf.
9 S. N. Omenyi, A. W. Neumann and C. J. van Oss, *Journal of Applied Physics*, 1981, **52**, 789–795.
10 Surface Texture in Relation to Adhesive Bonding, https://www.astm.org/stp45683s.html, (accessed December 31, 2021).
11 N. R. Demarquette, J. C. Moreira, R. N. Shimizu, M. Samara and M. R. Kamal, *J. Appl. Polym. Sci.*, 2002, **83**, 2201–2212.
12 J. Chen and J. A. Gardella, *Macromolecules*, 1998, **31**, 9328–9336.
13 D. J. Geveke and R. P. Danner, *Polym. Eng. Sci.*, 1991, **31**, 1527–1532.
14 E. Occhiello, M. Morra, P. Cinquina and F. Garbassi, *Polymer*, 1992, **33**, 3007–3015.
15 N. Cho, H.-L. Yip and A. K.-Y. Jen, *Appl. Phys. Lett.*, 2013, **102**, 233903.
16 C. M. Burns and W. N. Kim, *Polym. Eng. Sci.*, 1988, **28**, 1362–1372.
17 K. Misichronis, W. Wang, S. Cheng, Y. Wang, U. Shrestha, M. Dadmun, J. W. Mays and T. Saito, *RSC Adv.*, 2018, **8**, 5090–5098.
